# Pathophysiology of Cardiovascular Complications in COVID-19

**DOI:** 10.3389/fphys.2020.575600

**Published:** 2020-10-09

**Authors:** Vladimir Petrovic, Dina Radenkovic, Goran Radenkovic, Vukica Djordjevic, Maciej Banach

**Affiliations:** ^1^Department of Histology and Embryology, Faculty of Medicine, University of Nis, Nis, Serbia; ^2^Guy’s and St. Thomas’ Hospital and King’s College London, London, United Kingdom; ^3^Institute of Public Health, Department of Virology, Faculty of Medicine, University of Nis, Nis, Serbia; ^4^Polish Mother’s Memorial Hospital Research Institute (PMMHRI), Lodz, Poland

**Keywords:** COVID-19, CVD, cytokine release syndrome, thrombosis, Pediatric Inflammatory Multisystem Syndrome

## Abstract

Numerous recent studies have shown that patients with underlying cardiovascular disease (CVD) are at increased risk of more severe clinical course as well as mortality of COVID-19. Also, the available data suggests that COVID-19 is related to numerous *de novo* cardiovascular complications especially in the older population and those with pre-existing chronic cardiometabolic conditions. SARS-CoV-2 virus can cause acute cardiovascular injury, as well as increase the risk of chronic cardiovascular damage. As CVD seem to be the major comorbidity in critically unwell patients with COVID-19 and patients often die of cardiovascular complications, we review the literature and discuss the possible pathophysiology and molecular pathways driving these disease processes: cytokine release syndrome, RAAS system dysregulation, plaque destabilization and coagulation disorders with the aim to identify novel treatment targets. In addition, we review the pediatric population, the major cause of the cardiovascular complications is pediatric inflammatory multisystem syndrome that is believed to be associated with COVID-19 infection. Due to the increasingly recognized CVD damage in COVID-19, there is a need to establish clear clinical and follow-up protocols and to identify and treat possible comorbidities that may be risk factors for the development of cardiovascular complications.

## Introduction

Coronavirus disease 2019 (COVID-19) is a novel infection first documented in December 2019 in Wuhan, China (Zhou P. et al., 2020). The disease carries the risk of a severe acute respiratory syndrome coronavirus 2 (SARS-CoV-2) and its pathogenesis is still unknown. The spectrum of COVID-19 seems to lie from asymptomatic or mild viral illness to a systemic disease characterized by pneumonia, fever, dry cough, breathing difficulties (dyspnea), headache, anosmia and occasional diarrhea (Chen N. et al., 2020; Guan et al., 2020; Huang et al., 2020).

Numerous recent studies have shown that patients with underlying cardiovascular disease (CVD) are at increased risk of more severe clinical course of the disease, as well as mortality. Reports from Wuhan, Lombardy and New York showed that hypertension is the most common cardiovascular comorbidity among the patients admitted for hospital care (The Novel Coronavirus Pneumonia Emergency Response Epidemiology Team, 2020; Grasselli et al., 2020; Richardson et al., 2020). Diabetes and obesity, although not strictly CVD, were also identified as the predictors of severe clinical course in patients with COVID-19, regardless of age and sex (Kass et al., 2020; Kassir, 2020; Li et al., 2020). Also, the patients without the history of CVD were reported to develop cardiovascular complications during COVID-19 that may contribute to the bad outcome of the disease (Guo et al., 2020; Wang D. et al., 2020; Wu and McGoogan, 2020) and COVID-19 infection can raise cardiac biomarkers and cause direct cardiac and vascular injury (Bavishi et al., 2020). As CVD seem to be the major comorbidity in critically unwell patients with COVID-19 and patients often die of cardiovascular complications, we review the possible pathophysiology of these disease processes (Palmieri et al., 2020).

## COVID-19 and the Cardiovascular System

It is well documented that the influenza infections, as well as SARS and MERS viruses can cause cardiovascular complications that are most often represented in the form of myocarditis, acute myocardial infarction, acute heart failure, arrhythmia, sub-clinical diastolic impairment and cardiac arrest (Ferrari, 2020; Xiong et al., 2020). Like in previous coronavirus outbreaks, the available data suggests that COVID-19 is related to numerous cardiovascular complications especially in the older population and those who already have chronic conditions. SARS-CoV-2 virus can cause acute cardiovascular injury, as well as increase the risk of chronic cardiovascular damage (Zheng S. et al., 2020). Also, the patients with pre-existing cardiovascular conditions face increased risk of mortality when affected with the SARS-CoV-2 (Zheng Y.Y. et al., 2020). According to the analysis of Emami et al., that included 76993 patients presented in 10 studies, hypertension, CVDs, diabetes, kidney disease, smoking, and chronic obstructive pulmonary disease were among the most prevalent underlying diseases among hospitalized patients with COVID-19. Of these, CVD had the highest prevalence among diseases that put patients at higher risk from COVID-19 and was 12,11% (Emami et al., 2020). Based on a meta-analysis performed in China on 72,314 patient records, mortality in the group of the patients with CVD was shown to be 10.9% (The Novel Coronavirus Pneumonia Emergency Response Epidemiology Team, 2020). Initial reports from China pointed to the cardiovascular complications arising in patients affected with SARS-Cov-2. In a cohort study performed on first 41 admitted patients in Wuhan, China, Huang et al. report that 5 out of 41 patients (12%), with proven SARS-CoV-2 infection, have developed acute cardiovascular injury (ACI) during the hospital treatment. Four of these patients were admitted to the intensive care unit, due to the severe clinical picture. All of the patients with ACI had an increase in high-sensitivity cardiac troponin I. Further laboratory analyses, performed on all 41 patients, showed that prothrombin time and D-dimer level on admission were higher in ICU patients (median prothrombin time 12,2 s; median D-dimer level 2,4 mg/L) than non-ICU patients (median prothrombin time 10,7 s; median D-dimer level 0,5 mg/L) (Huang et al., 2020). In another study, performed on 138 hospitalized patients in Wuhan, China, 10 patients developed ACI, of which eight were transferred to ICU. Also, arrhythmia was evidenced in 23 patients of which 16 were transferred to ICU. The laboratory findings in patients with ICU showed a similar profile as in a previous study. All patients with ACI had increase in high-sensitivity cardiac troponin I. Laboratory analyses showed that prothrombin time and D-dimer levels were higher in ICU patients (respectively, 13,2 s and 414) compared to the non-ICU patients (12.9 s and 166, respectively) (Wang D. et al., 2020).

In a study performed on 416 patients in Wuhan, Shi et al. (2020) report that 82 patients (19.7%) had cardiac injury. Compared to the patients without cardiac injury, these patients were older, had more comorbidities, had higher leukocyte counts and levels of C-reactive protein, procalcitonin, creatine kinase–myocardial band, myohemoglobin, high-sensitivity troponin I, N-terminal pro-B-type natriuretic peptide, aspartate aminotransferase and creatinine. Greater proportions of patients with cardiac injury required non-invasive mechanical ventilation (46.3 vs 3.9%) or invasive mechanical ventilation (22.0 vs 4.2%) than those without cardiac injury. Complications were also more common in patients with cardiac injury than those without cardiac injury and included acute respiratory distress syndrome (58.5 vs 14.7%), acute kidney injury (8.5 vs 0.3%), and coagulation disorders (7.3 vs 1.8%). Patients with cardiac injury showed higher mortality rate than those without cardiac injury (51.2 vs 4.5%) (Shi et al., 2020).

Cardiovascular complications mostly occur in admitted severe or fatal cases of COVID-19, that have already developed ARDS. The risk of heart injury was higher in severe cases, approximately 22.2–31%, than in mild cases, approximately 2–4% (Zhao et al., 2020). Myocardial injury and acute coronary syndrome were seen as cardiovascular complications in patients with severe or fatal respiratory infection caused by SARS-CoV-2 and were the signs of poor prognosis (Driggin et al., 2020). Also, the cardiac arrythmias were commonly present as a cardiovascular complication in COVID-19 patients, especially in severe cases and in patients in ICU compared to the non-ICU cases (44,4 vs 6,9%) (Wang D. et al., 2020). Wang reports that 16,7% of patients develop cardiac arrythmias as a part of COVID-19 symptomatology (Wang D. et al., 2020). However, studies from China report that 7,3% of hospitalized patients (10/137) had heart palpitations as a first symptom of COVID-19 (Liu et al., 2020). In the restrospective cohort study that included 191 patients, Zhou reports that 23% of patients developed heart failure, which was more common in fatal cases compared to survivors (52 vs 12%) (Zhou F. et al., 2020). Interestingly, Dong reports the development of four end-stage heart failure as a cardiovascular complication in two male patients with mild COVID-19 infections (Dong et al., 2020).

However, studies performed in pediatric patients with COVID-19 with cardiovascular complications, report the laboratory profile (cardiac enzymes, coagulation status) being within the normal reference range (Chen J. et al., 2020; Wang Z. et al., 2020).

## Age and Sex Differences Related to Cardiovascular Complications in COVID-19

The overall data suggest that the patients above 60 years of age are at higher risk from development of cardiovascular complications. The data from China reveals that only 0,5% of patients in their 40 s died from COVID-19, while the death rate increases with age (3,6% in 60 s, 8% in 70 s and 15% in 80 s). The data from Italy show that the lethal outcome was seen in 25% of patients in their 70 s and 31% in their 80 s (Márquez et al., 2020). Reports from Italy showed that cardiovascular comorbidities were the most commonly associated with risk of death of COVID-19, most notably hypertension (70%), ishaemic heart disease (30%), atrial fibrillation (20%), and heart failure (15%) (Penna et al., 2020). It is interesting to notice that the male/female ratio of lethality is above 1.1, going to 1.7 in some countries such as Spain, Italy, England, Belgium, Greece, Denmark, and Netherlands (Márquez et al., 2020). There have been many speculative theories to explain for such differences including that women in middle age tend to have preferential lipid profile as compared to men of same age partly due to the protective effects of female hormones. Consequently, it is possible that CVD are more prevalent in men, and that patients with already existing CVD have higher risk of complications during COVID-19 infection (Penna et al., 2020). In addition, basic science research has shown that estrogens can up regulate the expression of ACE2 in the female heart tissue. This may increase the port of entry for the virus, but can significantly limit the subsequent inflammatory response and cytokine storm (Bukowska et al., 2017).

## Cytokine Release Syndrome and Haemodynamic Instability

Cytokine release syndrome (CRS) represents systemic inflammatory response that was described after the administration of the anti-T-cell antibody muromonab-CD3 (OKT3), a medication that was used as an immunosuppressant after organ transplantation (Chatenoud et al., 1990; Shimabukuro-Vornhagen et al., 2018). CRS, as a form of innate immune response, can also occur as a complication of viral infections and is responsible for ARDS (acute respiratory distress syndrome) and multiple organ failure (Shimabukuro-Vornhagen et al., 2018). The cases of CRS outbreaks were reported during previous MERS and SARS epidemics, and there are also reports evidencing CRS in patients affected with SARS-CoV-2 (Hu et al., 2017; Xu et al., 2020).

Clinically, CRS can present in mild or more severe forms. Mild forms include fever, fatigue, rash, myalgia, artralgia, that are often seen at the onset of many infectious diseases and cannot be easily distinguished from other viral illnesses. However, CRS can progress to a more severe form, severe inflammatory response (SIRS), that manifests with hemodynamic compromise resulting in circulatory shock, vascular leakage, DIC (disseminated intravascular coagulopathy), and multisystem organ failure – cardiac dysfunction, renal and hepatic failure and ARDS (Murthy et al., 2019). The laboratory parameters usually show cytopenia, elevated levels of C-reactive protein, deranged markers of coagulation and thrombosis (D-dimer, prothrombin time) and the deranged levels of organ specific markers (Shimabukuro-Vornhagen et al., 2018).

Cardiac function is also affected in CRS which manifests mostly as a cardiomyopathy that resembles the one seen in sepsis and stress cardiomyopathy (Takotsubo cardiomyopathy). The cardiovascular complications during CRS arise as a result of acute cardiac toxicity and their pathogenesis is not completely understood (Lee et al., 2014). The ejection fraction can be reduced, and the other symptoms of cardiac dysfunction include the arrythmias, hypotension and tachycardia. The acute cardiac failure and elevated levels of troponin indicating acute cardiac injury can also appear as a complication of CRS (Lee et al., 2014; Shimabukuro-Vornhagen et al., 2018). Laboratory analyses of COVID-19 patients show that lymphopenia is connected with the severe clinical presentation. The number of T lymphocytes (CD4+, CD8+), B lymphocytes, NK cells, as well as the eosinophils, basophils and monocytes, is reduced, and the number of neutrophils usually shows higher percentage value (Cao, 2020).

**FIGURE 1:**
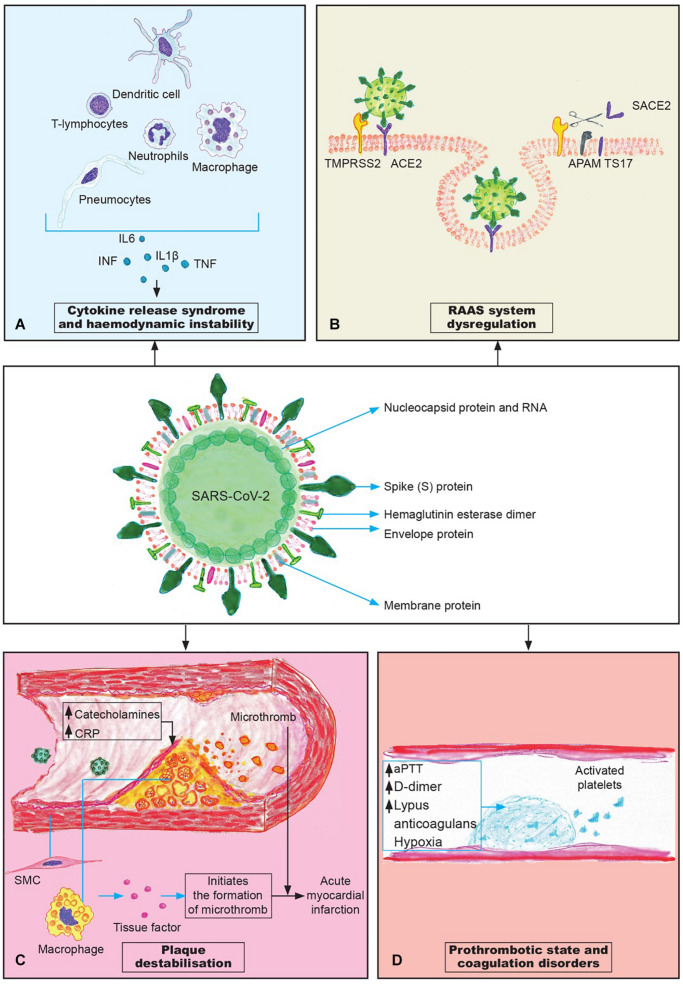
The pathogenesis mechanisms of cardiovascular complications in COVID-19. **(A)** Cytokine release syndrome (“cytokine storm”) is a systemic inflammatory response that plays the crucial role in the development of cardiovascular complications; **(B)** The downregulation of ACE2 receptors; **(C)** Plaque destabilization can lead to the plaque rupture and consequent formation of microthrombi; **(D)** Coagulation disorders contribute to the cloth formation and microthrombosis in different organs.

CRS represents dysregulated and excessive immune response that fails to defend the organism against the infection, and instead damages the body. In their detailed review on CRS in SARS and COVID-19, Ye et al. (2020) discuss that the respiratory epithelial cells, dendritic cells and macrophages, after infection with SARS-CoV, firstly produce low levels of cytokines and chemokines, and in the later stages these cells secrete low levels of the antiviral factors interferons (IFNs) and high levels of proinflammatory cytokines: IL-1β, IL-6, and tumor necrosis factor (TNF), as well as the chemokines CCL-2, CCL-3, and CCL-5 (Figure 1A). The increased levels of cytokines and chemokines attract inflammatory cells, such as neutrophils and monocytes that convert to macrophages, which results in massive infiltration of the lung tissue causing lung injury, but also the parenchyma of other organs may be affected as well (Ye et al., 2020). In the lungs, the massive infiltration of neutrophils and macrophages causes diffuse alveolar damage with the formation of hyaline membranes and a diffuse thickening of the alveolar wall, that leads to ARDS (Cao, 2020). IFN-α/β (interferon-α/β) or macrophage-derived proinflammatory cytokines induce the apoptosis of T cells, which promotes further rapid viral replication, and cause the apoptosis of respiratory epithelial and endothelial cells (Hogner et al., 2013; Channappanavar et al., 2016). Apoptosis of endothelial cells and epithelial cells damages the pulmonary microvascular and alveolar epithelial cell barriers and causes increased endothelial permeability and alveolar edema.

Patients with COVID-19 have shown to have increased levels of IL-1B, IFN-γ, IP-10, and monocyte chemoattractant protein 1 (MCP-1) that are part Th1 immune response responsible the activation of specific immunity, but also higher levels of IL-4 and IL-10 that represent the part of the TH2 immune response that shows the anti-inflammatory effects (Ye et al., 2020). Also, IL-1β and TNFα (highly expressed by TH17 and TH1 cells), both promote TH17 responses and increased endothelial permeability leading to the vascular leakage (Wu and Yang, 2020).

The studies have shown that the level of IL-6 correlates positively with the severity of the disease and the outcome, and that the use of drugs that inhibit the secretion of IL-6 may have an important role in the treatment of CRS (Henderson et al., 2020; McGonagle et al., 2020). The other potential targets in the treatment of CRS could be IL-1 and IL-17, and the future clinical trials are needed to show the effectiveness and benefit of developing the drugs targeting their receptors and molecules involved in their signaling pathways (Cao, 2020).

## RAAS System Dysregulation

The mechanisms underlying the cardiovascular complications in patients with SARS-CoV-2 infection are still not completely elucidated. ACE2 (angiotensin-converting enzyme-2), as the point of entry of SARS-CoV-2 virus into the cell, is currently in the focus of researchers, especially bearing in mind their wide distribution on pneumocytes type 2 and endothelial cells of the arteries, arterioles and venules in various organs, including the lungs and the heart (Oudit et al., 2009).

ACE2 is a single-pass type I transmembrane metallocarboxypeptidase, with its enzymatically active domain exposed on the cell surface (Hamming et al., 2004). ACE2 functions as a negative regulator of the RAAS (renin-angiotensin-aldosteron system) and has the role in degradation of angiotensin-2 which results in the production of the heptapeptid called angiotensin 1–7 (Crackower et al., 2002). Angiotensin 1–7 binds to G-protein coupled mas oncogene receptor, exerting the vasodilatator, anti-hypertrophic and anti-inflammatory effects on the cardiovascular system (Crackower et al., 2002; Der Sarkissian et al., 2008; Zhong et al., 2010). On the other side, angiotensin-2, as a part of RAAS, binds to its receptors – angiotensin-2 receptors type 1 (AT1) and causes vasoconstriction that leads to the rise of blood pressure. If overstimulated, angiotensin-2 can negatively impact the cardiovascular system, by causing hypertension, inflammation, myocardial fibrosis and hypertrophy that all eventually lead to heart failure (Mehta and Griendling, 2007). The loss of ACE2 has been documented to have a negative impact on the cardiovascular system, leading to the cardiac hypertrophy and contractility disorders (Kassiri et al., 2009; Patel et al., 2012). Patel et al. (2014) reported that higher concentrations of angiotensin-2 suppress ACE2 by increasing TNF-α converting enzyme (TACE) activity which leads to the cleavage of the extracellular portion of ACE2, thus rendering the rest of the protein ineffective (Figure 1B). The cleavage of active sites results in the elevated plasma levels of ACE2 which is considered to be a marker of disease and poor prognosis (Bitker and Burrell, 2019). The downregulation of ACE2 was also documented in lungs especially in the men during ageing, in diabetes mellitus and is considered to be one of the factors causing hypertension (Verdecchia et al., 2020).

Upon the entrance into the cell via ACE2 receptor, SARS-CoV-2 leads to the downregulation of these receptors (Zhang H. et al., 2020). This newly established imbalance between ACE2 and angiotensin-2 may cause the appearance of cardiovascular complications in patients with no previous history of CVD or worsen the existing CVD in patients with COVID-19. The downregulation of ACE2 in mice experimental model that occurs after the infection with SARS-CoV worsens acute lung failure *in vivo* that can be attributed to the effects of angiotensin-2. The use of angiotensin-2 receptor blockers attenuates this situation by inhibiting the activity of RAAS (Kuba et al., 2005). Study performed on a murine model by Oudit et al. showed that SARS-CoV can affect the heart, leading to the partial down-regulation of *Ace2* mRNA expression with a complete loss of myocardial ACE2 protein levels (Oudit et al., 2009). The results obtained from the autopsies of the hearts of patients affected with SARS-CoV-2 showed the pronounced decrease in the ACE2 protein levels and macrophage infiltration. These data may suggest that the downregulation of protective function of ACE2 might cause myocardial inflammation and damage leading to the myocardial dysfunction (Oudit et al., 2009).

Although ACE2 serves as a receptor for SARS-CoV-2, its blockage would have a negative impact on the health of the patient due to its array of biological roles. However, in the study performed by Bertram et al. (2012) it was reported that the modest ACE2 expression in the upper respiratory tract might limit SARS-CoV transmissibility. Although SARS-CoV and SARS-CoV2 share many similarities, it is important to notice that SARS-CoV-2 has a furin cleavage site at the S_1_/S_2_ boundary, which is believed to increase its transmissibility and/or altering its pathogenicity compared to SARS-CoV that does not possess this sequence (Walls et al., 2020). At this moment, when the vaccine is still in development, one of the possible treatment options would be camostate mesylate, an inhibitor of enzyme TMPRSS2 that plays a crucial role beside ACE2 in the entry of the virus into the cell (Hoffmann et al., 2020). Also, the sera from the patients who recovered from SARS infections showed the ability to cross-neutralize SARS-CoV-2 entry into the cell (Hoffmann et al., 2020).

ACE inhibitors and ARBs are the widely used medications in the treatment of arterial hypertension and in prevention of heart remodeling. There is still not sufficient data concerning the effects of ACE inhibitors and ARBs on the levels of ACE2 in humans (Park et al., 2020; Vaduganathan et al., 2020). Although there were some suggestions that the use of ACE inhibitors and angiotensin receptor blockers (ARB) may facilitate the entry of the virus in the cells, the current recommendation of European Society of Cardiology is that the patients using these drugs in the therapy of hypertension should not discontinue their usage (European society of cardiology). Some authors discuss the potential beneficial effects of ARBs in the therapy lung injury in COVID-19 patients with hypertension (Gurwitz, 2020; Phadke and Saunik, 2020; Verdecchia et al., 2020). This is based on the ability of these medications to suppress angiotensin-2 effects on worsening the inflammation of the lung parenchyma during SARS-CoV-2 infection and to elevate the levels of downregulated ACE2 that produces the angiotensin 1–7. There are currently announced trials aiming to examine the role of losartan as a supporting therapy in patients with COVID-19. Observational studies until now show a survival benefit in patients with ACE2 inhibitors (Verdecchia et al., 2020).

## Plaque Destabilisation

The increased level of catecholamines, which was shown to occur in COVID-19, as part of systemic inflammation may lead to the plaque rupture and destabilization, thus causing acute coronary syndrome (Basu-Ray et al., 2020). Also, the levels of C-reactive protein were shown to be in the direct correlation with the risk of the onset of myocardial infarction due to plaque rupture (Ridker et al., 1997). Wang reported that C-reactive protein is elevated in patients with COVID-19, and that its levels correlate with the severity of clinical presentation (Wang, 2020).

The rupture of atheromatous plaque leads to the exposure of foamy macrophages, located under the endothelium, to the bloodstream. These macrophages express the tissue factor that, in contact with the blood, initiates the formation of microthrombi. Also, the rupture of the plaque exposes vascular smooth muscle cells to the blood flow, which also expresses tissue factor that facilitates the process of thrombogenesis (Libby and Simon, 2001; Figure 1C). Additionally, smooth muscle cells may undergo inflammatory activation which results in the excessive production of IL-6 that can induce the acute phase response. Therefore, some of the commonly prescribed drugs for lipid-lowering therapy like statins that are also believed to have plaque stabilizing properties might prove to be beneficial in a subgroup of COVID-19 patients with pre-existing atherosclerotic disease (Bahrami et al., 2018).

## Prothrombotic State and Coagulation Disorders

The systemic inflammation caused by different infectious agents is also known to be associated with the disturbances in the hematopoietic system (Connors and Levy, 2020). It has the pro-coagulant effect thus facilitating the formation of microthrombi, which consecutively may cause the infarction of different organs. The disturbances in the coagulation were evidenced in the early reports from Wuhan, China. In a study that included 99 patients in Wuhan, China, Chen N. et al. (2020) reported significantly higher values for activated partial thromboplastin time (6% of the patients), prothrombin time (5%) and higher levels of D-dimer (36%). However, studies performed by Wang D. et al. (2020) (138 patients) and Huang (41 patients), showed minimal increase of values for prothrombin, prothrombin time and D-dimer (Huang et al., 2020).

In a study performed in Wuhan on 189 patients, it was reported that 21 patients (11,5%), who died during the hospital treatment, showed significantly higher levels of D-dimer and fibrin degradation products, and longer prothrombin time and activated partial tromboplastin time (aPTT) compared to survivors. Also, they reported that 71,4% of non-survivors developed disseminated intravascular coagulation (Tang et al., 2020).

In a study comprising 216 COVID-19 positive patients, Bowles et al. (2020) measured the coagulation status parameters. They reported that 44 (20%) patients had prolonged aPTT time. Further analyses showed that 34 out of these 44 patients were positive on lupus anticoagulant assays (Bowles et al., 2020). Lupus anticoagulant are antibodies that belong to the group of antiphospholipid antibodies and are associated with a thrombotic tendency within the antiphospholipid syndrome. They are directed against the anionic phospholipids or other membrane particles that are exposed to the immune system after membrane remodeling that occurs under different infectious, inflammatory or autoimmune stimuli (Gebhart et al., 2019; Helms et al., 2020). The role in pathogenesis if COVID-19 needs to be further elucidated (Bowles et al., 2020).

Helms et al. (2020) in a study performed on 150 patients diagnosed with COVID-19, report that more than 95% of patients had elevated D-dimer and fibrinogen. Results of their analysis show that Von Willebrand (vWF) activity, vWF antigen and FVIII (factor VIII) were considerably increased, and that 50/57 tested patients (87.7%) had positive lupus anticoagulant. 16,7% of patients developed pulmonary embolism during their hospital treatment, and there were no cases of DIC.

High D-dimer in admitted patients was usually associated with poor prognosis and a high mortality rate. Also, worsening lymphopenia over time with increased levels of D-dimer were the findings mostly seen in non-survivors (Basu-Ray et al., 2020).

The activation of coagulation during the systemic inflammation can occur through several mechanisms. Polyphosphates derived from microorganisms activate platelets, mast cells and factor XII of coagulation. Also, the system of complement and components of NETs (neutrophil extracellular traps), as well as the pathogen associated molecular mechanisms are involved in the activation of coagulation cascade (Connors and Levy, 2020). All the above-mentioned shows that the activation of coagulation cascade during the systemic inflammatory response is highly complex and involves several mechanisms that simultaneously lead to the formation of microthrombi and possible development of DIC. The procoagulant effects of hypoxemia should be also considered in the pathogenesis of coagulation disorders in COVID-19, bearing in mind that patients affected with this disease may develop a drop in oxygen saturation. The low levels of oxygen activate the transcription factor Egr-1 (early growth response-1) that leads to the transcription and translation of tissue factor in mononuclear phagocytes and smooth muscle cells, which results in the vascular fibrin deposition. Also, hypoxia upregulates plasminogen activator inhibitor-1 which suppresses fibrinolysis (Yan et al., 1999). The induction of hypoxia induced factor and endothelial inflammation during hypoxia and systemic inflammatory response may contribute to the formation of microthrombi (Chang, 2019; Gupta et al., 2019; Figure 1D).

## Pediatric Inflammatory Multisystem Syndrome in Children

The pediatric population seems to be less affected with COVID-19 compared to the older individuals. The report show that only 1–2% of children were diagnosed with COVID-19 and that the risk of lethal outcome is 500 times lower than in adults (Fischer, 2020). There is still ongoing discussion concerning the mild clinical picture of COVID-19 in children. Several factors are being accounted for in the discussion, including the prevalence of antibodies against seasonal coronaviruses in children, expression of ACE2 and the recent previous vaccination with a live vaccine such as BCG vaccine for Tuberculosis (Felsenstein and Hedrich, 2020). However, we still don’t know the exact molecular mechanisms protecting children from severe outcomes of COVID-19 infection. Pagliaro proposes that the macrophage heterogeneity might be one of the protective key elements in children, discussing that the macrophages enter the lungs in three developmental waves of which two occur *in utero*, while the third one happens after birth. He further discusses that these three waves give rise to the three different lineages that differ among themselves by the expression of cell surface markers including ACE2 and that children most probably have more of macrophages that came through the first two waves of migration (Pagliaro, 2020). The role of macrophages is important for the initiation of innate immune response after infection with SARS-CoV-2, which occurs trough the activation of the NF-kappaB pathway (Chang et al., 2020). These three lineages of macrophages populate human lungs and their relation changes over time with ageing. Each one of these three types of macrophages may elicit different responses to virus infection thus possibly giving the different clinical pictures in children and in adults (Pagliaro, 2020).

There have been recent reports of children and adolescents developing Paediatric Inflammatory Multisystem Syndrome (PIMS) associated with COVID-19, that has a similar clinical picture to Kawasaki disease (Cheung et al., 2020; Jones et al., 2020; Riphagen et al., 2020; Whittaker et al., 2020). Kawasaki disease (KD) is a medium-vessel disease vasculitis that often affects the coronary arteries and is characterized by an acute onset, cardiac complications and self-limiting course (World Health Organization, 2020). The disease is more common in East Asian pediatric population (Newburger et al., 2004). As KD might affect coronary arteries, it can cause arrhythmia, acute heart failure and hemodynamic instability, a condition known as Kawasaki disease shock syndrome (KDSS) (Kanegaye et al., 2009).

The exact pathogenesis of KD is still unknown. There are reports that viruses from coronavirus family might be associated with the development of KD, however these results need further verification (Shirato et al., 2014; Verdoni et al., 2020). The studies report that the majority of children affected with PIMS were not having active COVID-19 infection, but IgG and IgM antibodies against SARS-CoV-2 were elevated in laboratory findings (Whittaker et al., 2020). This may lead to the assumption that the possible cause of PIMS might be the aberrant delayed immune response triggered by COVID-19 infection (Cheung et al., 2020; Whittaker et al., 2020). Also, that IL-6, IL-10 and interferon-gamma that are elevated in KD as well as in COVID-19, where these are shown to exert an inflammatory-mediated lung injury (Li et al., 2019; Cheung et al., 2020).

The treatment of PIMS has been mostly with aspirin and intravenous immunoglobulin (IVIG) sometimes co-administered with antibiotics to prevent super-infection, which is the treatment for KD, mostly due to the similar clinical picture. Although IVIG was widely used in the treatment of severe cases of SARS-CoV-2 in adults, there is still not enough evidence about its effectiveness in the treatment of children with PIMS (Zhang J. et al., 2020).

## COVID-19 Pandemic Societal Effect as a Cardiovascular Risk Factor

Lastly, with the COVID-19 pandemic response lasting for longer than 6 months in many countries all over the globe, one cannot exclude the indirect effects such as social isolation, chronic stress, change in eating habits and, for many, reduced physical activity with stay-at-home measures potentially contributing to cardiovascular risk in patients with preexisting conditions.

In the aim of dealing with the COVID-19 pandemic, the majority of countries have at some point implemented the total or partial quarantine of the population. Many mental health specialists have alerted already that the “lock down” phenomenon led to the raise of the number of patients with anxiety and higher stress levels, but also caused the changes in the lifestyle especially concerning the eating habits and lower physical activity (Mattioli et al., 2020). The chronic stress, perhaps arising as a consequence of the quarantine and social isolation or on the other hand, fear of political and financial aftermath, could lead to increase of the activity of the sympathetic nervous system which may cause increased risk of mortality in patients with pre-existing CVD (Steptoe and Kivimäki, 2012; Mattioli et al., 2020). In a study that was following a cohort of 1267 patients older than 65 years in a period of 10 years, it was shown that the social isolation was highly connected with the increased risk of mortality in these patients (Yu et al., 2020).

The “2019 ACC/AHA Guideline on the Primary Prevention of CVD” recommended that the adults should engage in at least 150 min per week of accumulated moderate-intensity or 75 min per week of vigorous-intensity aerobic physical activity, but those who cannot meet the required minimum should be anyway engaged in the moderate or vigorous physical activity even if it is less than recommended (Arnett et al., 2019). The physical activity plays a crucial role in reduction of inflammation and oxidative stress, but it is also important in maintaining the normal weight, thus decreasing the risk of diabetes mellitus, CVD and metabolic disorders (World Health Organization, 2018). Bearing in mind that the gyms and public spaces were closed during the quarantine hours it is recommended that the people should be involved in in-house physical activity in order to prevent the possible negative health impacts caused by the sedentary way of life. These considerations have rightly so not been the top priority in the immediate pandemic response. Nevertheless, as the world continues to battle this pandemic while awaiting for treatments and a vaccine, it is important to take consideration the general health and wellbeing of the population should there be reinstitution of quarantine measures especially in the autumn/winter months with often worse weather conditions for outdoor exercise in the Northern hemisphere. Although healthy lifestyle promotion is unfortunately often missed in the busy medical consultations, public health interventions for the prevention of CVD should become a fundamental tool in the pandemic response.

## Perspective and Future Directions

There is a need to define the most important comorbidities related to the cardiovascular system that can contribute to the development of the disease, in order to address the attention of medical personnel on possible complications. Also, the psychological counzelling should be provided to patients in order to make sure that they are aware of the reality of the situation and to prepare them to better cope with their hospital staying and post COVID-19 follow-up (Dolinski et al., 2020).

Patients with hypertension, diabetes and/or obesity represent high-risk group that should be closely monitored in order to prevent or properly treat the possible complications due to the SARS-CoV-2 infection. Of special interest are men and patients older than 60 years with severe disease, who were shown to have a longer duration of virus in stool, serum and respiratory samples (Zheng S. et al., 2020). As thromboembolic events tend to occur at higher frequency in patients with pre-existing atherosclerosis in COVID-19 infections, higher risk groups should be appropriately given anticoagulation therapy if in the hospital. Potentially high risk groups with pre-existing CVD disease even in milder confirmed COVID-19 could be evaluated by blood tests including cardiac specific markers and lipid levels, as well as electrocardiography and echocardiography to further risk stratify patients who would benefit from lipid lowering, plaque stabilizing and anti-platelet or anti-coagulation medications without an unacceptable risk of bleeding.

The regular therapy for CVD should not be discontinued during COVID-19 infection (Guzik et al., 2020). As it is increasingly recognized there is a direct damage to the blood vessels and the heart, there have been reports of the use of standard drugs for CVD prevention in COVID-19. ACE inhibitors may have a potential beneficial role. Statins and lipid lowering drugs have been suggested for the treatment of patients with COVID-19 due to their ability to disrupt lipid rafts in the cell membrane and prevent the binding of the virus on the cell. In patients with CVD their usage should not be discontinued. However, it remains unclear whether starting these medications prophylactically or during COVID-19 has any clinical benefit (Banach et al., 2020; Katsiki et al., 2020; Reiner et al., 2020).

Currently, we have limited knowledge on the possible cardiovascular complications that may arise in the aftermath of COVID-19 infection. There has only so far been speculative work about the molecular effects on the cardiomyocytes and endothelial cells and a possible increased risk of heart failure that might represent later. However, due to the increasingly recognized CVD damage in this disease, we will need to have a longer term follow up of severe COVID-19 patient survivors to answer this question.

## Author Contributions

DR conceived the concept of the manuscript. All authors contributed to the literature review and writing of the manuscript and approved the final version.

## Conflict of Interest

The authors declare that the research was conducted in the absence of any commercial or financial relationships that could be construed as a potential conflict of interest.
